# Solid Form and Phase Transformation Properties of Fexofenadine Hydrochloride during Wet Granulation Process

**DOI:** 10.3390/pharmaceutics13060802

**Published:** 2021-05-27

**Authors:** Suye Li, Hengqian Wu, Yanna Zhao, Ruiyan Zhang, Zhengping Wang, Jun Han

**Affiliations:** 1 College of Chemistry, Chemical Engineering and Materials Science, Shandong Normal University, Jinan 250014, China; suyeli@stu.sdnu.edu.cn; 2 School of Chemistry and Chemical Engineering, University of Jinan, Jinan 250022, China; hengqianwu@mail.ujn.edu.cn; 3 Institute of BioPharmaceutical Research, Liaocheng University, Liaocheng 252000, China; zhaoyanna@lcu.edu.cn (Y.Z.); zhangruiyan@lcu.edu.cn (R.Z.); wangzhengping@lcu.edu.cn (Z.W.); 4 Liaocheng High-Tech Biotechnology Co. Ltd., Liaocheng 252000, China

**Keywords:** fexofenadine hydrochloride, phase transition, wet granulation, water content, granule characterization

## Abstract

The quality control of drug products during manufacturing processes is important, particularly the presence of different polymorphic forms in active pharmaceutical ingredients (APIs) during production, which could affect the performance of the formulated products. The objective of this study was to investigate the phase transformation of fexofenadine hydrochloride (FXD) and its influence on the quality and performance of the drug. Water addition was key controlling factor for the polymorphic conversion from Form I to Form II (hydrate) during the wet granulation process of FXD. Water-induced phase transformation of FXD was studied and quantified with XRD and thermal analysis. When FXD was mixed with water, it rapidly converted to Form II, while the conversion is retarded when FXD is formulated with excipients. In addition, the conversion was totally inhibited when the water content was <15% *w*/*w*. The relationship between phase transformation and water content was studied at the small scale, and it was also applicable for the scale-up during wet granulation. The effect of phase transition on the FXD tablet performance was investigated by evaluating granule characterization and dissolution behavior. It was shown that, during the transition, the dissolved FXD acted as a binder to improve the properties of granules, such as density and flowability. However, if the water was over added, it can lead to the incomplete release of the FXD during dissolution. In order to balance the quality attributes and the dissolution of granules, the phase transition of FXD and the water amount added should be controlled during wet granulation.

## 1. Introduction

Polymorphism widely exists in active pharmaceutical ingredients (APIs), and it may occur during the formulation or manufacturing processes. Wet granulation is the most commonly used process in the pharmaceutical industry for achieving agglomeration of the primary powder particles into a workable granule with a solvent or liquid binder [1]. The main objective of the process is to improve the material properties, such as improved flowability and compression, improved uniform drug distribution and content uniformity, reduced dustiness and minimized segregation [2,3]. During binder addition, the formulation components go through a series of process, including wetting and nucleation, growth and consolidation, together with attrition and breakage [1]. However, in these processes, active ingredients or excipients undergo dissolution, phase transformation and recrystallization, which can alter physiochemical properties, including the solubility, stability, hygroscopicity, particle size and shape, flowability and mechanical behaviors etc. [4,5]. For example, Zhang et al. [6] reported eight crystals of acotiamide hydrochloride, corresponding interestingly to different stability and solubility. Silva et al. [7] described mebendazole existed three polymorphs with different solubility. What is more, the polymorphs can affect drug product quality and performance. For instance, polymorph A does not present the desired effect when it exceeds 30% in a formulation due to its low solubility. Crystal polymorphism, if occurring during manufacturing, may affect the physical properties and, consequently, the efficacy and safety of the drug products. Jacon Freitas et al. [8] stressed the importance of the polymorphic characterization, because the presence of a small amount of MLX Form III significantly affected the rate of dissolution of tablets. Barakh Ali et al. [9] described the presence of dihydrate crystal of carbamazepine during manufacturing leading to clinical failure and change in dissolution characteristics. It is thus necessary to strictly control phase transformation during manufacturing. Although widely used, wet granulation is also most likely to cause phase transformation due to the addition of solute [10]. The understanding and control of processes have been paid more and more attention by regulatory agencies which has resulted in the ICH tripartite guideline Q6A: “Test Procedures and Acceptance Criteria for New Drug Substances and New Drug Products: Chemical Substances” [11]. Hence, it is necessary to determine whether phase transformation occurs during processing and ideally gain a mechanistic understanding that will improve formulation design and guide the proper selection of process parameters to enable control, leading to a robust process [12].

During wet granulation process, the addition of water or solution in the formulation can improve the properties of the powder, but is also likely to induce phase transformation of API and excipients. Meanwhile, the binder impacts many of the granule properties such as hardness, disintegration time and physiochemical properties of active ingredients [13].Thus, the amount of water or solution required is an important parameter which has to be taken into account during wet granulation. Dharti Tank et al. investigated the effect of solvents on thermal properties of microcrystalline cellulose granules [2]. Based on thermal effusivity measurements, the binder concentration was determined. Some previous studies used excipient—microcrystalline cellulose as model to determine the end-point of wet granulation [2,14,15]. Similarly, for APIs that undergo phase transformation during wet granulation progress, the extent of phase transformation of API can also be used as a tool to select an appropriate water addition and evaluate the granulation endpoint.

Fexofenadine hydrochloride, (±)-4-[1 hydroxy-4-[4-(hydroxydiphenylmethyl)-1-piperidinyl]-butyl]-α, α-dimethyl benzeneacetic acid hydrochloride, is a white to off-white crystalline powder and a third-generation antihistamine drug. It can competitively and reversibly inhibit the H_1_ receptor [16]. It is originally marketed under the trade name Allegra^TM^, which is one of the blockbusters in the section of antihistamines to treat allergic coryza, idiopathic urticaria and similar diseases [17]. In the development of generic drugs of Fexofenadine hydrochloride, phase transformation was observed during the wet granulation process in presence of water. As a result, fexofenadine hydrochloride was utilized as a model drug to investigate the relationship between water content and the degree of phase transformation in wet granulation process by analyzing its thermal behavior properties in this paper. The properties of fexofenadine hydrochloride were studied with thermogravimetric analysis (TGA), differential scanning calorimetry (DSC), infrared spectroscopy (IR) and powder X-ray diffraction (PXRD). In addition to the research regularity of phase transformation, the influence of phase transformation was also understood through granules characterization and dissolution experiments.

## 2. Experimental Section

### 2.1. Materials

**Figure 1:**
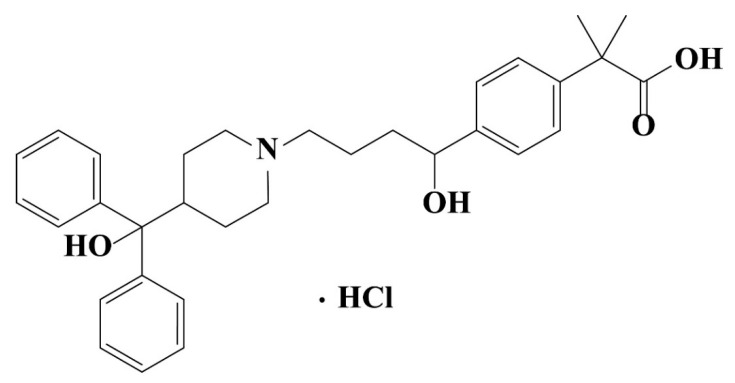
Chemical structure of fexofenadine hydrochloride (FXD).

Fexofenadine Hydrochloride (FXD, C_32_H_40_ClNO_4_, Figure 1) was purchased from Ind-Swift Laboratories Limited (Chandigarh, India) without further purification and identified as Form I by PXRD. The ultrapure water was prepared by Milli-Q^®^ Advantage A10^®^ water purification system (Millipore, Billerica, MA, USA, electrical resistivity was 18.2 MΩ·cm at 25 °C). Hydrochloric acid and sodium hydroxide were purchased from Yantai Far Eastern Fine Chemical Co. Ltd (Yantai, China). HPLC grade acetonitrile was obtained from Shanghai Titan scientific Co., Ltd. (Shanghai, China). HPLC grade triethylamine, acetic acid and phosphoric acid were obtained from Fisher Scientific (Thermo Fisher Scientific, Waltham, MA, USA). All other experimental excipients are presented in Table 1.

### 2.2. Instrumentation

Thermogravimetric analysis (TGA) was recorded using a Discovery-TGA instrument (TA instruments, New Castle, DE, USA). TGA traces were recorded at a heating rate of 10 °C/min under a nitrogen purge of 60 mL/min. Sample with masses between 4 to 8 mg were analyzed using platinum high temperature pan.

The thermal behavior was analyzed by differential scanning calorimetry (DSC) using Discovery DSC^TM^ (TA instruments, New Castle, DE, USA). Temperature and enthalpy were calibrated with indium standard. The sample (2–5 mg) was weighted by an MSA6-6S analytical balance with an uncertainty of 0.001 mg (Satorius, Göttingen, Germany), then placed in aluminum pan and scanned at ramping rate of 10 °C/min under a dry nitrogen atmosphere with a flow rate of 200 mL/min. The heating and cooling ramps of cyclic DSC curves were as follows: Ramp 1 = heating rate of 10 °C/min from 30 to 220 °C; Ramp 2 = cooling rate of 10 °C/min from 220 to −40 °C; Ramp 3 = heating rate of 10 °C/min from −40 to 220 °C. The data were analyzed by TRIOS software and the drawing was generated with OriginPro 9.0 (OriginLab Corporation, Northampton, MA, USA).

Powder X-ray diffraction (PXRD) patterns were obtained with Ultima IV X-ray diffractometer (Rigaku, Japan) with a CuKα radiation (1.541836 Å) at room temperature. The tube voltage and current were set at 40 kV and 40 mA, respectively. The divergence slit and anti-scattering slit settings were set at 0.5° for the illumination on the 10 mm sample size. Sample was measured by a continuous scan between 3° and 40° in 2θ with a step size of 0.02°. The experimental PXRD patterns were refined using OriginPro 9.0 (OriginLab Corporation, Northampton, MA, USA).

Fourier transforms infrared (FTIR) spectra was collected by a Nicolet 6700 Fourier-Transform Infrared Spectroscopy spectrometer (Thermo Scientific, Waltham, MA, USA) using Potassium Bromide pellets technique in the region 500–4000 cm^−1^. The experimental data was analyzed and refined using OriginPro 9.0.

Karl Fischer Titration (KFT) was used to confirm the final water content of sample by wet-granulation process. The water content in the sample (~30 mg) was determined by coulometric Karl Fischer titration (851 Titrando, Metrohm, Herisau, Switzerland).

### 2.3. Characterization of FXD Polymorphs

FXD Form I is the starting material used in a wet granulation for making tablets. FXD exhibits polymorphic conversion during the wet granulation process. The pure phase-transformed form was prepared via the form conversion in the wet granulation process. Further drying was carried out under vacuum at 60 °C for 24 h. The characterization of phase-transformed form was analyzed by TGA, DSC, FTIR and PXRD. The effect of the high temperature and humidity environment on pure phase-transformed form was investigated. The pure phase-transformed form was placed into an uncapped glass vials and monitored at 60 °C, 92.5% RH at room temperature for 30 days. Then the samples were analyzed by PXRD at 0, 5, 30 days.

### 2.4. Setting of the Granulation Process

#### 2.4.1. Apparatus

A certain amount of drug and excipients were pre-mixed and granulated with the addition of water in a high shear granulator (FHSG 20, Foryou Mechatronics Ltd., Shanghai, China). The high shear granulator was equipped with a spray system (Spraying Systems Co., Glendale Heights, IL, USA) including spray nozzles and pressure pot to make the water stay spray pattern and spray evenly on the materials. The pressure of the spraying system was 0.3 MPa. The final water content of wet mass was confirmed by Karl Fischer Titration. The water content of sample by wet-granulation process was selected as the input variables instead of addition of water (mg). Then, wet mass with different water content was tray dried at 60 °C for fully dried in electro-thermostatic blast oven and dried granules were collected for further analysis.

#### 2.4.2. Preparation of FXD Wet Mass

To assess the influence of the water content on phase transformation behavior of pure FXD, FXD (150 g) was stirred and granulated into a 2 L wet granulator and wet mass of FXD with different water contents (0%, 5%, 10%, 15%, and 20%) were prepared. In the granulation process, the stirring paddle and cutter were set at 150 rpm and 500 rpm, respectively. Then, wet mass was sampled and the water content measured by KFT shall prevail. The wet mass with different water content was dried and collected for further analysis.

#### 2.4.3. Preparation of Formulation Wet Mass

A total of 200 g of formulation powder was pre-mixed and granulated with the addition of water in a 2 L high shear granulator, containing 40% FXD, 17% MCC, 40% PGS, and 3.0% CCNa. The stirring paddle and cutter were set at 100 and 250 rpm, respectively. The powder was mixed and sprayed with water during granulation. The wet mass with different water contents 0%, 5%, 10%, 15%, 20%, 25%, 30%, 35%, and 40% *w*/*w* were prepared. Then, wet mass with different water content was put into a vacuum oven at 60 °C for fully dried and dried granules were collected for DSC analysis. The wet mass before and after drying step was analyzed by PXRD. It is worth mentioning that the use of excess amounts of water in wet-granulation process did not reflect a realistic formulation composition but was used for measurement under extreme condition. This extreme method provides insight into the effect of water content on phase transformation.

For validation of the relationship between phase transformation of FXD and water content, laboratory scale capacities of 454.2 g formulation were granulated in 4 L granulator, while the same composition of the formulation was maintained in wet granulation process. The stirring paddle and cutter were set at 250 and 500 rpm, respectively. The API and excipients were pre-mixed and granulated with the addition of water by spraying. Validation samples containing 0%, 5%, 10%, 15%, 20%, 25%, 30%, 35%, and 40% *w*/*w* were also prepared and analyzed using the same procedure.

#### 2.4.4. Data Analysis of the Degree of Phase Transformation of FXD

The FXD Form I % content at different water contents was calculated using Equation (1). The Form I (%) was plotted against water content.

(1) $$\mathrm{Form}\;I\;(\%)=\frac{\Delta H_{i\;}}{\Delta H_{0\;}}$$

where Δ*H*_0_ represents the melting enthalpy of pure FXD (72.0 J/g) and is used as a reference. In granulation of pure FXD, Δ*H*_i_ represents the melting enthalpy of the sample determined by DSC analysis. In granulation of formulation, Δ*H*_i_ is defined as quotient of the melting enthalpy of the sample determined by DSC analysis to the FXD content (%, *w*/*w*) of FXD-excipients formulation. In other words, the enthalpy Δ*H*_i_ value has been normalized for the FXD content in formulation [18,19]. The quantification of Form I content % can be used to reflect the phase transformation of FXD during wet granulation process.

### 2.5. Study on Effect of Phase Transformation

#### 2.5.1. Polarized Light Microscope Observation

A small amount of FXD were placed on a slide glass and covered with another slide glass. A medium was then penetrated between the slides by capillary action from the side of the slide glass using a micropipette [20,21]. The dissolution and precipitation processes after contact with medium (about 20 μL) were observed under a Axio Scope. A1 polarized light microscope (Zeiss, Oberkochen, Germany). Pure water, 0.01 mol/L HCl and 0.01 mol/L NaOH were used as media in order to investigate the influence of different pH and ionic strength on the dissolution and precipitation processes of FXD.

#### 2.5.2. Preparation of Different Media-Based Granule

The granules were prepared using 200 g formulation powder containing 40% FXD (Form I), 17% MCC, 40% PGS, and 3.0% CCNa in a 2 L high shear granulator with 0.01 mol/L HCl, pure water and 0.01 mol/L NaOH of all three media. The stirring paddle and cutter were set at 100 and 250 rpm, respectively. The same technological procedure was used across all of the granulation process, containing the same composition and weight of the formulation, the media amount (0.01 mol/L HCl, pure water and 0.01 mol/L NaOH) and addition method. Then, the wet mass granules were passed through sieve #30 and dried at 60 °C for 3 h. After drying, different media-based granule was subjected to thermal and granule characterization.

#### 2.5.3. Granule Characterization

Angle of repose: A sample of granules was dispensed through a funnel to form a conical heap, with fixed height (2 cm) of the cone and the radius *r* of cone circular measured to calculate the angle of repose (AOR) using the following equation. The procedure was repeated thrice, with each formulation granule, and results averaged [22].

AOR = tan^−1^ (h/r)

Compressibility test: The bulk density (D_B_) of granules was measured using graduated cylinder. The weight of granules and untapped volume of the packing were then recorded and its D_B_ was defined as quotient of weight to volume of the powder mass. The tapped volume (V_T_) of packing was performed using graduated cylinder at the free-falling condition under gravity to get a constant value. The tapped density (D_T_) was expressed as the weight to volume ratio of the same mass after tapping. The results are the mean of at least triplicates. The Hausner ratio and Carr index are then defined as the ratio and relative difference between the tap density and the untapped density, respectively [23,24,25].

CI% (Compressibility index) = 100 (V_B_ − V_T_)/V_B_

CrI% (Carr index) = 100 (D_T_ − D_B_)/D_T_

HR (Hausner ratio)= D_T_/D_B_

Hardness: In experimental studies, the flow properties of granules have shown direct correlation with the hardness of tablets manufactured [26]. In order to compare the hardness difference of each media-based granule, the same weight (100 mg) of a single tablet was individually added to each 6-mm punch hole to produce using Rotary tablet press (ZP-9, Liaocheng Tianchi Pharmaceutical Machinery Co., Ltd., Liaocheng, China) with constant compression force. Hardness of the tablet of each media-based granule was measured by using hardness tester (YD-35, Tianjin Tianda Tianfa Technology Co., Ltd., Tianjin, China). For each batch, six tablets were selected randomly and evaluated.

Particle size: The particle size and size distribution for each formulation granule was obtained by laser diffraction measurement (Mastersizer 3000, Malvern Instruments, Worcestershire, UK) via the dry dispersion method following standard test procedures. The particle size D (10), D (50) and D (90) were determined. A total of six replicates were conducted for each formulation granule and the results averaged [25].

#### 2.5.4. Determination of Drug Content

Appropriate weight (~100 mg) of media-based granules equivalent to 40 mg of FXD was placed into a 50 mL volumetric flask. 50 mL of acetonitrile −0.17% acetic acid mixture (3:1, *v*/*v*) was then added and shaken mechanically until the granules were dissolved. A portion of this solution was filtered, and further diluted with acetonitrile −0.17% acetic acid mixture (3:1, *v*/*v*) to a concentration of 80 µg/mL. The samples were analyzed by HPLC with DAD-3000RS detector (Thermo Fisher Scientific, Waltham, MA, USA) at a wavelength of 220 nm. Chromatographic separation was performed at 35 °C using a Phenyl column (Inertsil^®^ Ph-3, 5 μm, 4.6 × 250 mm, GL Sciences Inc., Tokyo, Japan). The mobile phase was consisted of acetonitrile: mobile phase B (36:64 *v*/*v*). The mobile phase B: acetonitrile-triethylamine (1:1) 15 mL was diluted to 1 L with 0.17% acetic acid, and pH was adjusted to 5.25 ± 0.05 with phosphoric acid. The samples were monitored at a flow rate of mobile phase 1.5 mL/min and injected with a volume of 20 µL.

#### 2.5.5. Dissolution

Dissolution tests of 3 tablets of each media-based granule was performed on dissolution tester (Distek dissolution apparatus Model symphony 7100, Serial number 7101251, North Brunswick, NJ, USA) by paddle method. An accurately weighed quantity of the tablet was dispersed in the vessel containing 900 mL of dissolution medium (distilled water, 37 °C) with a stirred speed of 50 rpm. Since the solubility of FXD in distilled water was 2.7 ± 0.02 mg/mL at 37 °C (reported solubility at 25 °C: 1.5 ± 0.02 mg/mL [27]), sink condition could be maintained. Aliquots (4.5 mL) were taken at predetermined time points of 5, 10, 15, 20, 30, 45, 60, 90, 120 min and followed by addition of an equal volume of fresh dissolution media. The samples were filtered with 0.45 µm polyether sulfone (PES) syringe filter to HPLC vials and analyzed by HPLC with UV detection.

## 3. Results and Discussion

### 3.1. Solid State Characterization of Polymorphs

**Figure 2:**
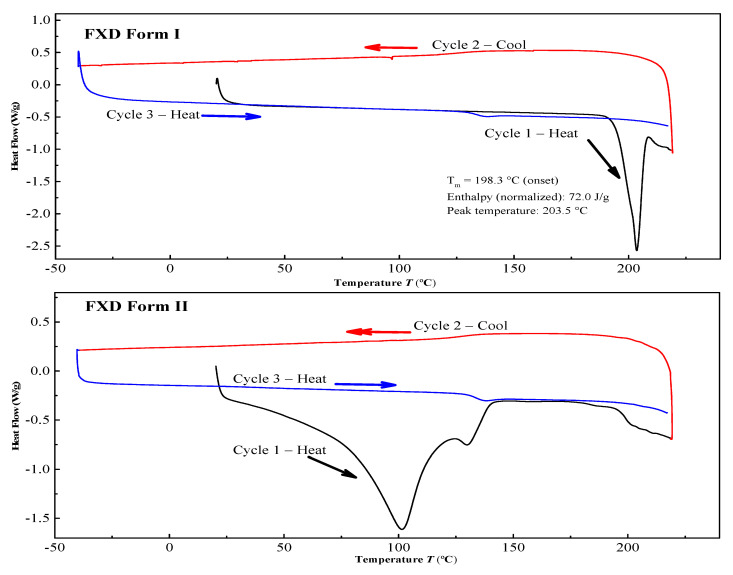
Differential scanning calorimetry (DSC) curves of the FXD solid forms.

**Figure 3:**
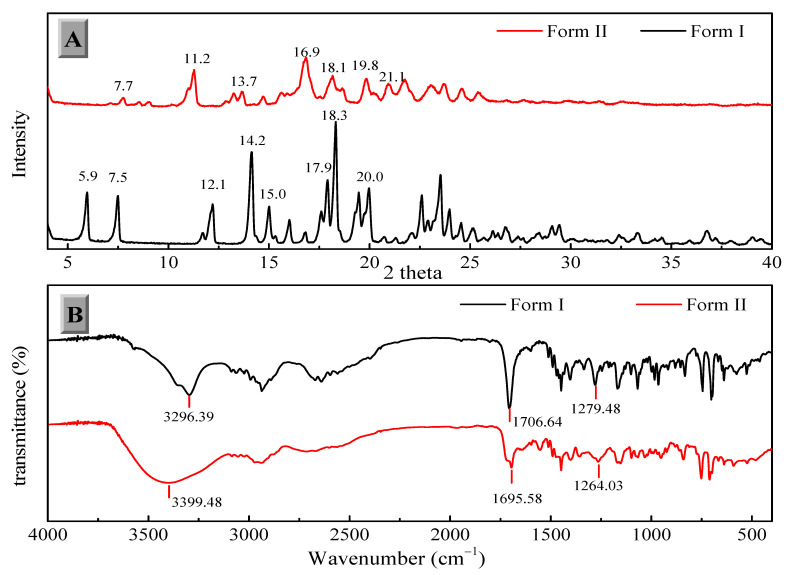
Powder X-ray diffraction (PXRD) (**A**) and Fourier transforms infrared (FTIR) spectroscopy (**B**) of the FXD solid forms.

The two solid forms of FXD were characterized by DSC, PXRD and FT-IR. The DSC thermograms of FXD solid forms are shown in Figure 2. The FXD Form I showed a sharp melting peak at 198.3 °C (onset T), as well as the value of its enthalpy (72.0 J/g), which was in good agreement with the reported melting point values of hydrochloride salt FXD Form I in the range of 193–199 °C. While phase-transformed form showed a dehydration peak at about 100 °C and a small endothermic event at 128 °C (onset T) (endotherm peak 132 °C), which corresponded with the reported endothermic peak of hydrochloride salt FXD Form II (hydrate) in the range of 124–126 °C [28]. The number of water molecules in phase-transformed solid form was estimated based on TGA analysis. Phase-transformed solid form showed a total weight loss of 3.5% before decomposition, which was indicative of this form as a monohydrate crystalline form (calculated value: 3.23%) (Supplementary Figure S1). PXRD shown in Figure 3A was used to characterize the two solid forms of FXD. The characteristic peaks of Form I were at 2θ = 5.9°, 7.5°, 12.1°, 14.2°, 15.0°, 17.9, 18.3°, and 20.0°; and the characteristic peaks of Form II were at 2θ = 7.7°, 11.2°, 13.7°, 16.9°, 18.1°, 19.8°, and 21.1° [28,29]. The FTIR spectra of two solid forms were presented in Figure 3B. The characteristic peaks of FXD Form I was observed at 3296.39 cm^−1^ (OH stretching), 1706.64 cm^−1^ (C=O carboxylic acid stretching) and 1279.48 cm^−1^ (CN stretching of tertiary amine). The major difference in the FTIR spectra of the two forms of FXD was observed as OH symmetrical stretching at 3399.48 cm^−1^ and several shifts in the fingerprint region between 400 cm^−1^ and 1800 cm^−1^. All these observations showed good agreement with the values reported in the literature [30,31]. By compared with the PXRD and FTIR results reported in the literature, it was confirmed that phase-transformed solid form of FXD was crystalline Form II. The comparison data of the characterization with relevant data published in the literature were listed in detail in Table S1. In addition, Form II thermogram displayed a new and unique endothermic peak at 132 °C without melting peak of Form I. Besides, there was no disturbance of excipients melting events at the point of 198.3 °C. Based on the pronounced difference at 198.3 °C, it was possible to quantify FXD solid forms (Form I) according to the difference in the melting enthalpy.

### 3.2. Granulation Process

#### 3.2.1. Water-Induced Phase Transformation of FXD without Excipients

**Figure 4:**
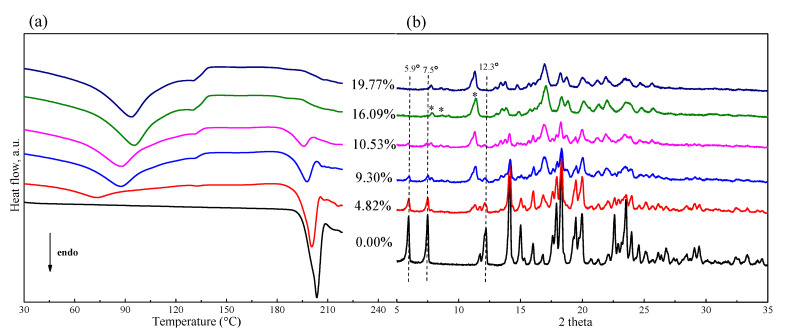
DSC thermogram (**a**) and PXRD patterns (**b**) of FXD–water physical mixtures by wet-granulation process with different water contents. The (*) pointed the appearance of characteristic peaks for Form II with the increase of water content.

It was found that the extent of phase transformation of FXD was related to water content. To determine the relationship, a preliminary study for FXD alone by wet-granulation process was carried out with DSC and the results are shown in Figure 4a. In the presence of water, an immediate conversion of the FXD from Form I to Form II occurred. With the increase of water content, the melting peak of FXD disappeared gradually, when water content was ≥16% *w*/*w*, the peak almost completely disappeared due to the conversion of FXD from Form I to Form II. In addition, in a physical mixture of FXD with ~5% *w*/*w* water content, a weak inflection or exothermic change at ≈ 132 °C appeared. The weak inflection at ≈132 °C transformed into a broad peak and had an obvious increase with the increase of water content. These results provided direct evidence of water-induced phase transformation. As shown in Table 2, the enthalpy (Δ*H*), an indicator of amount of FXD Form I, was decreased by the addition of the water.

The corresponding PXRD curves are visually presented in Figure 4b and provided additional insight about phase transformation in wet granulation of FXD alone. The diffractogram of FXD showed a decrease in the intensities of characteristic peak, which were pointed out by dotted vertical lines; it was accompanied by the appearance of new peaks which were pointed out (*) with the increase of water content. From PXRD peak analysis, it can be concluded that the crystal form of FXD at water content ≥ 16% *w*/*w* is not identical with it original from, and the crystal form is a polymorphic mixture at water content > 0% *w*/*w* and <16% *w*/*w*. The PXRD results supported the DSC results for the effect of water content on phase transformation of FXD.

#### 3.2.2. Water-Induced Phase Transformation of FXD with Excipients

**Figure 5:**
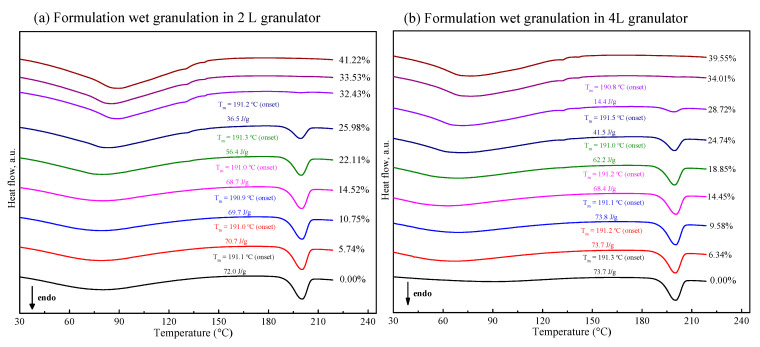
DSC thermogram of tablet formulation by wet-granulation process with different water contents in 2 L (**a**) and 4 L (**b**) granulators. The enthalpy values have been normalized for the FXD content in formulation.

In order to comprehensively understand the effect of water content on polymorphism behavior of FXD in tablet formulation, the mixtures containing FXD and excipients was processed by wet granulation with different water content in 2 L granulator. Figure 5a shows the DSC thermogram of granules formed at the end of the granulation. In the range of low water content (<10% *w*/*w*), the Δ*H* of FXD Form I did not have a pronounced variation. However, when the water content was more than 15% *w*/*w*, and the Δ*H* decreased obviously. Unlike the phase transformation behavior of the FXD alone, the FXD in formulation revealed a slow conversion from Form I to Form II, which was presumably due to the hygroscopicity of excipients.

**Figure 6:**
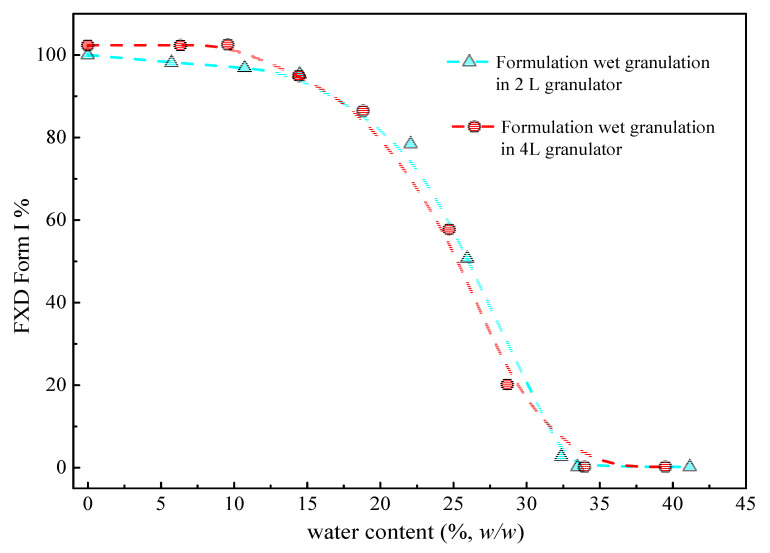
Percentage of FXD Form I as a function of water content in 2 L and 4 L granulators by DSC during wet granulation formulation. The line is drawn to assist in visualizing the trend.

Figure 6 (cyan line) illustrates the phase transformation of FXD Form I as a function of the water content for wet granulations in 2 L granulator. It can be seen that the extent of phase transformation depends on the amount of water added. An increase of transformation rate started at >15% *w*/*w* water content, and 50% of Form I can be obtained when the water content reached to 25% *w*/*w*. Furthermore, the complete phase transformation was observed when the water content was 35% *w*/*w*. Herein, we confirmed the relationship between the percentage of Form I FXD and water content during phase transformation. The effect of the scale-up and water addition amount were studied at the 4 L granulator scale and the results presented in Figure 6 (red line). It can be seen that the effects of water content on Form I content (%) are similar between 4 and 2 L granulator scales. Doubling the apparatus does not influence the relationship between the phase transformation and water content. The amount of water added is considered as the most critical parameter that influences the granulation process and determines properties of the resultant granules [32]. In this study, it was shown that the granulation endpoint can be evaluated using the extent of phase transformation of FXD. The relationship between phase transformation and water content can be used to select the appropriate amount of water and finalize the process parameters.

#### 3.2.3. Effect of Drying

**Figure 7:**
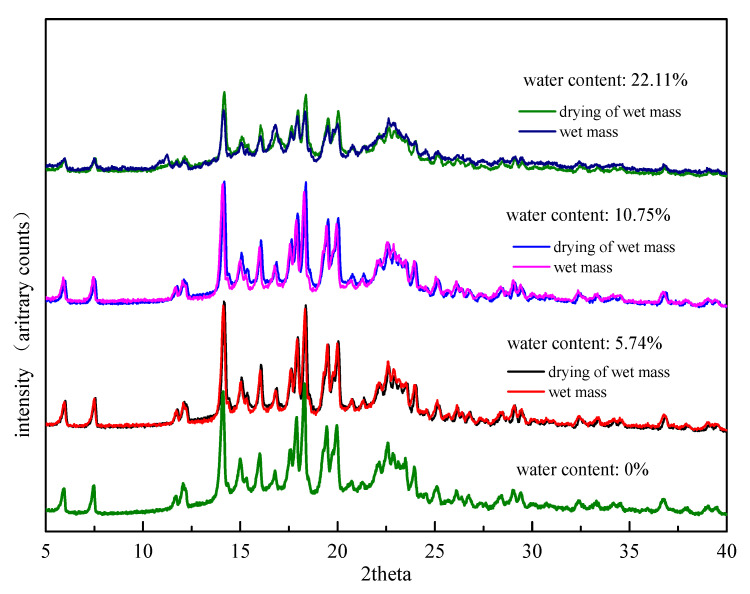
PXRD patterns of formulation wet mass with different water contents before and after drying at 60 °C.

The drying of the wet mass at 60 °C is potentially a critical step. The stability of Form II was monitored at 60 °C and 92.5% RH at room temperature for 30 days. The results of PXRD measurements kept consistent with the original Form II, which demonstrated that Form II was stable without secondary phase transition under 60 °C (Supplementary Figure S2). The influence of the drying step on the degree of drug transformation was examined. In the preparation process of formulation wet mass, the wet mass with different water contents were immediately collected and PXRD was applied before and after the drying step of wet mass. The PXRD results were presented in Figure 7. The relative peak intensities of FXD Form I before and after drying were close to one another under low water content conditions (5.74%, 10.75%) suggesting that comparatively small changes were observed after drying. The drying step appeared to lock the final degree of drug transformation during wet granulation. In contrast, the relative peak intensities of FXD Form I after drying was obviously higher than that of wet mass at high water content (22.11%). This phenomenon indicated that phase conversion of wet mass under high water content continued before drying. However, the timely drying step appeared to retard, even interrupt the continuation of phase transformation. In a word, the timely drying step was a favorable influence factor to reflect the true degree of phase transformation.

### 3.3. The Effect of Phase Transformation on the FXD Tablet Performance

Previously, some literatures reported that the phase transformation occurred in active ingredients or excipients could affect the final product performance [8,33,34,35]. Therefore, in addition to identify the existence of phase transformation, it is also important to determine how it affects product performance.

#### 3.3.1. Polarized Light Microscope Observation of Dissolution and Precipitation of FXD

**Figure 8:**
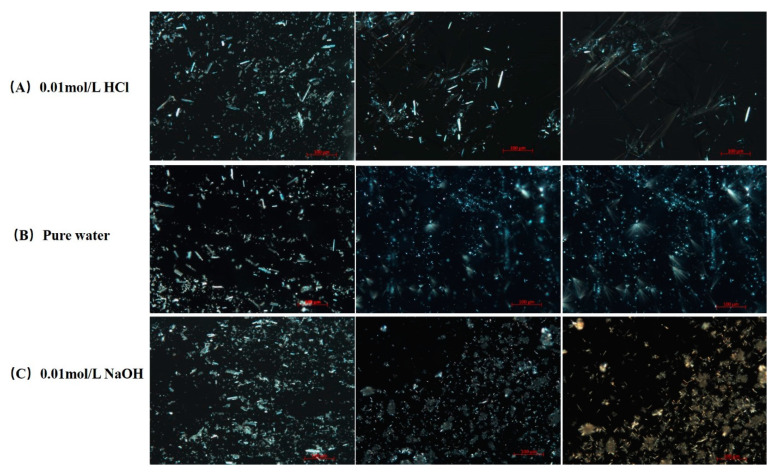
PLM images of dissolution and precipitation of pure FXD in various dissolution media under 200× magnification. (**A**) 0.01 mol/L HCl, (**B**) pure water, (**C**) 0.01 mol/L NaOH. Full pixel pictures are available in the Supplementary Information Figures S4–S6.

The dissolution and precipitation processes of FXD particles after contacting with various media were observed under a PLM and the images were presented in Figure 8. The same volume of 20 μL medium was penetrated between the slides by capillary action to initiate the dissolution of FXD particles. As shown in Figure 8, crystalline precipitates like sharp needle appeared with aggregation when contacted with 0.01 mol/L HCl and pure water medium. Needle-like crystals in 0.01 mol/L HCl medium grew faster than those in pure water medium. However, needle-like crystals without aggregation appeared when contacted with 0.01 mol/L NaOH medium. As PLM observations continued, a small number of needle-like precipitates with aggregation were observed. Based on the dissolution and precipitates observation of pure FXD under PLM, it can be inferred that the phase transformation of FXD during wet granulation is related to the needle-like with aggregation precipitates formation. Most active ingredients and fillers lack the binding ability to form a coherent mass, so it is necessary to add a binder, such as HPMC, to ensure excellent adhesion and cohesion between interparticulate surfaces in the wet state [36]. However, the slurry of FXD was observed having a degree of wet adhesion property. In addition, the formulation of FXD with excipients can be granulated successfully with only water. Given this phenomenon, it was speculated that phase-transformed FXD acted as binder, which enabled the formation of agglomerates and overcame unfavorable powder flow properties. However, with the change of water pH, it was observed that the binding properties are different.

#### 3.3.2. Thermal Behavior of Granule

**Figure 9:**
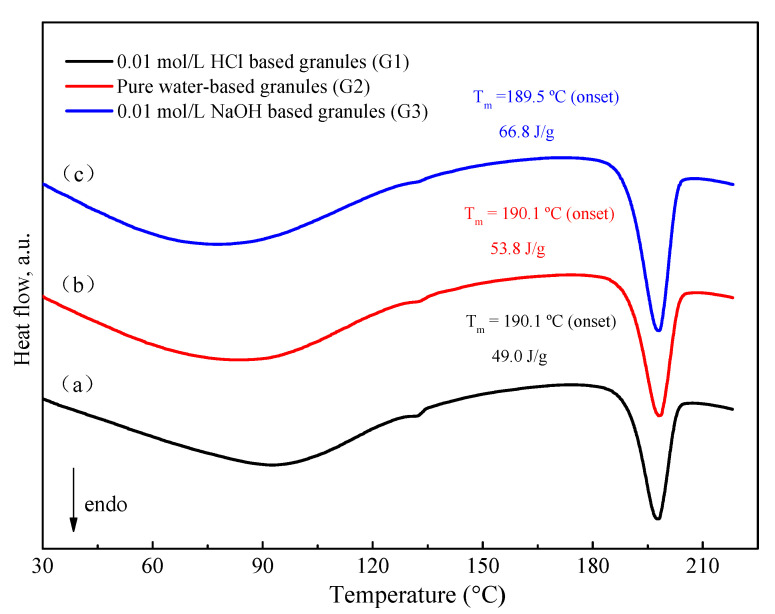
DSC thermogram of granule by wet-granulation process with various solvents. (**a**) 0.01 mol/L HCl, (**b**) pure water, (**c**) 0.01 mol/L NaOH. The enthalpy values have been normalized for the FXD content in formulation.

DSC thermograms for various media-based granules were obtained by the same technological procedure which was mentioned above. As shown in Figure 9, 0.01 mol/L HCl-based granules have relatively lower enthalpy values as compared to water-based and 0.01 mol/L NaOH-based granules. The enthalpy values directly correlate with the FXD Form I content. In other words, the order of the extent of phase transformation is: 0.01 mol/L HCl-based granules (G1) > water-based granules (G2) >> 0.01 mol/L NaOH-based granules (G3). Interestingly, this order is consistent with the growth rate of FXD needle-like precipitation in these solvents.

#### 3.3.3. Granule Characterization

**Figure 10:**
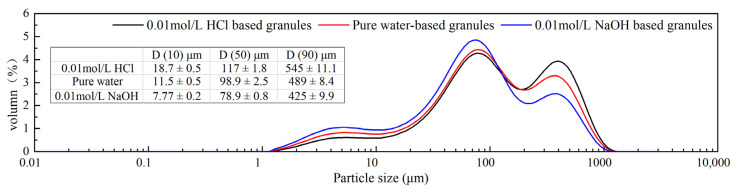
Different media-based granule particle size and size distribution.

The characterization of different media-based granules is shown in Table 3. In our study, the traditional flowability index (Hausner ratio, Carr’s index) and angle of repose were used to evaluate three types of media-based granules. The flowability of granules followed the order of 0.01 mol/L HCl-based granules > water-based granules > 0.01 mol/L NaOH-based granules. As expected, the results confirmed that the high extent of phase transformation contributed to the better flowability (Figure 10). In addition, the tablet hardness data were ranked in the following sequence: Tablets formulated using 0.01 mol/L HCl-based granules > water-based granules > 0.01 mol/L NaOH-based granules.

During wet granulation process, the formulation components go through a series of processes, including wetting and nucleation, dissolution, phase transformation together with recrystallization. In the process of preparing different media-based granules (0.01 mol/L HCl, pure water and 0.01 mol/L NaOH), the same technological procedure was used across all the granulation process. The solubility of the FXD was measured using “shake flask method” (150 rpm, 37 °C, 24 h) in different pH media. The solubility results were presented in Supplementary material Figure S3. Some literature overviews on FXD dissolution properties were reported [27,37,38]. The solubility and dissolution results indicated that 0.01 mol/L HCl-based granules dissolved to a higher extent than water or 0.01 mol/L NaOH media. In addition, we observed that the resulting solution after solubility measurement was filtered and left at room temperature, then crystalline precipitates like sharp needle appeared and grew faster to form a suspension. The suspension was observed having a degree of wet adhesion property. This may indicate that FXD go through dissolution and recrystallization during wet granulation process, which has a benefic effect on granulation. The changed technological properties of granule may be actually result from the changed amount of dissolved FXD during wet granulation process, which was difficult to be determined directly due to the presence of excipients. However, the phase transformation degree of FXD was positively correlated with the amount of dissolved FXD in granulation process. In addition, in this study, the phase transformation was determined by DSC. Therefore, an indirect relationship between the degree of phase transformation and the quality attributes of granules was established. Combined with the discussion above, the results showed that the high phase transition degree of FXD corresponded to the good flowability of particles.

#### 3.3.4. Drug Content and Dissolution Test

The drug content in media-based granules considering the quantity of powders equivalent to 40 mg FXD was determined as 100.11 ± 2.03% for 0.01 mol/L HCl-based granules, 99.86 ± 1.79% for 0.01 mol/L NaOH-based granules, and 97.17 ± 1.46% for water-based granules.

**Figure 11:**
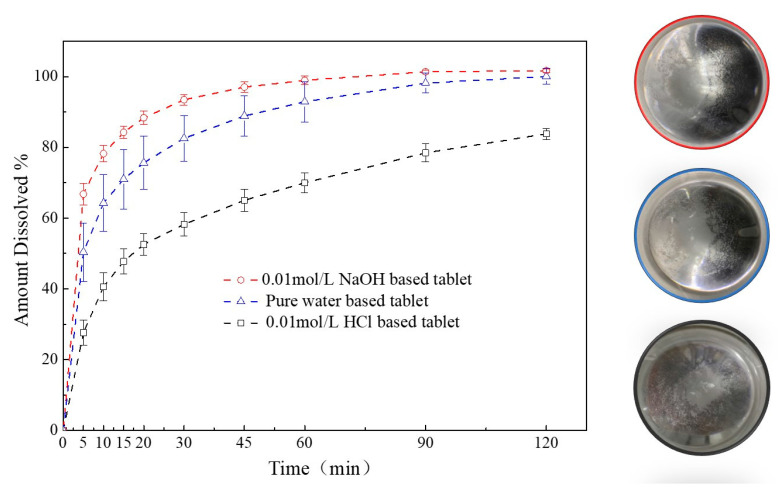
Comparison of dissolution profiles of different media-based granule formulated tablets.

A comparison of the dissolution profiles for different media-based granules formulated tablets were shown in Figure 11. The release of FXD from 0.01 mol/L HCl-based granules formulated tablets was less than 85% in 120 min, which was much lower than those from the pure water-based and 0.01 mol/L NaOH-based tablets. In contrast, within 90 min, FXD released completely from the pure water-based and 0.01 mol/L NaOH-based tablets. The FXD release rate increased significantly from the 0.01 mol/L NaOH-based tablets compared with the pure water-based tablet, because of the relatively lower phase transformation of FXD, that were consistent with the thermal behavior of granules study (Figure 9). In addition, after the dissolution experiment, the particles at the bottom of dissolution vessel were observed and agglomerates were found abundant in vessel of 0.01 mol/L HCl-based tablet, a small amount in the pure water-based tablet vessel, and none in 0.01 mol/L NaOH-based tablet vessel. This was aligned with the granule characterization study (Section 3.3.3) that FXD phase transformation played a crucial role in the formation of agglomerates during wet granulation process.

These results showed the effect of phase transformation of FXD on the dissolution rate for the different media-based tablets. Although the flowability of the particles was improved after phase transformation occurred, the high degree of phase transformation will lead to the consequence that the drug cannot be completely released from the tablets. Thus, the extent of phase transformation should be controlled within a certain range to balance the quality attributes of granules and dissolution of tablets.

## 4. Conclusions

In this study, water caused the phase transformation of FXD, while the presence of excipients slowed the conversion of FXD from Form I to Form II. The relationship between percentage of FXD Form I and water content was determined and proven to have a good correlation during wet granulation process. In addition, the study of phase transformation of FXD and the granule characterization of various media-based granules showed that the order of flowability of granules was consistent with that of the extent of phase transformation, being 0.01 mol/L HCl-based granules > water-based granules > 0.01 mol/L NaOH-based granules. The high phase transition degree of FXD corresponded to the good flowability of particles. It was also found that during the transition, the dissolved FXD acted as binder to improve the properties of granules, such as density and flowability. The pH of the binding solution would have an impact on the binding properties. The phase transformation degree of FXD was correlated with the amount of dissolved FXD in granulation process. However, the dissolution profile results for different media-based granules formulated tablets revealed that the high degree of phase transformation would lead to the incomplete release of the drug. Therefore, phase transformation of FXD in the wet granulation process is an important and measurable parameter that needs to be controlled within a certain range to balance the quality attributes and the dissolution of granules. Moreover, the relationship between phase transformation of FXD and water content can be used to guide process production, determine the appropriate amount of added water in the wet granulation process. The thermal behavior measurements tool could be combined with quality by design (QbD) to conduct process control, guarantee the consistency of product quality, and manufacture products with efficiency. The knowledge gained from this study around the impact of phase transformation on the manufacturing process has reference value for analogous drugs that undergo phase transformation during wet granulation process.

## Figures and Tables

**Table 1:** The list of ready-to-use excipients.

Chemical Name	Source	Lot Number
Fexofenadine hydrochloride (FXD)	Ind-Swift Laboratories Limited, Chandigarh, India	H007049003
Microcrystalline cellulose (MCC)	FMC Corporation, philadelphia, PA, USA	P115828088
Pregelatinized starch (PGS)	Colorcon, philadelphia, PA, USA	IN539042
Croscarmellose sodium (CCNa)	FMC Corporation, philadelphia, PA, USA	TN16829944

**Table 2:** Thermal characterization of FXD by wet-granulation process.

Water Content, % *w*/*w*	Crystallization Temperature		Δ*H*, J/g ^a^	Form I %
	Onset, °C	Peak, °C		
FXD				
0.00	198.3	203.5	72.0	100.0
4.82	196.2	200.5	53.1	73.7
9.30	189.6	197.6	26.2	36.3
10.53	187.3	195.5	13.2	18.3
16.09	^b^			0.0
19.77	^b^			0.0

^a^ The enthalpy value has been normalized for the FXD content. ^b^ No melting peak.

**Table 3:** Results of flow properties (mean ± SD, n = 3), hardness of tablets (mean ± SD, n = 6).

Parameter	0.01mol/L HCl-Based Granules	Pure Water-Based Granules	0.01mol/L NaOH-Based Granules
D_B_ (mg/mL)	0.38 ± 0.002	0.37 ± 0.009	0.35 ± 0.004
D_T_ (mg/mL)	0.50 ± 0.01	0.50 ± 0.02	0.51 ± 0.02
CrI (%)	24.61 ± 1.76	26.69 ± 4.29	30.4 ± 1.88
CI (%)	24.61 ± 1.76	26.69 ± 4.29	30.4 ± 1.88
HR	1.33 ± 0.03	1.37 ± 0.08	1.44 ± 0.04
Angle of repose (degrees)	45.5 ± 1.81	46.23 ± 0.46	49.63 ± 1.44
Weight (mg)	102.14 ± 2.17	102.83 ± 1.47	102.17 ± 3.13
Hardness (kgf)	12.74 ± 0.78	11.11 ± 2.05	10.05 ± 1.09

D_B_—bulk density; D_T_—tapped density; CrI—Carr index; CI—compressibility index; HR—Hausner ratio.

## Data Availability

Not applicable.

## Supplementary Materials

The following are available online at https://www.mdpi.com/article/10.3390/pharmaceutics13060802/s1, Table S1: The comparison data with relevant data published in the literatures or patents, Figure S1: Thermogravimetric analysis of the FXD solid forms, Figure S2: The stability study results of Form II, Figure S3: Histogram showing the study of equilibrium solubility of FXD in different media, Figure S4-1: PLM images of dissolution and precipitation of pure FXD in (A) 0.01 mol/L HCl media under 200× magnification (the left figure in the first row), Figure S4-2: PLM images of dissolution and precipitation of pure FXD in (A) 0.01 mol/L HCl media under 200× magnification (the middle figure in the first row), Figure S4-3: PLM images of dissolution and precipitation of pure FXD in (A) 0.01 mol/L HCl media under 200× magnification (the right figure in the first row), Figure S5-1: PLM images of dissolution and precipitation of pure FXD in (B) pure water media under 200× magnification (the left figure in the second row); Figure S5-2: PLM images of dissolution and precipitation of pure FXD in (B) pure water media under 200× magnification (the middle figure in the second row), Figure S5-3: PLM images of dissolution and precipitation of pure FXD in (B) pure water media under 200× magnification (the right figure in the second row), Figure S6-1: PLM images of dissolution and precipitation of pure FXD in (C) 0.01 mol/L NaOH media under 200× magnification (the left figure in the third row), Figure S6-2: PLM images of dissolution and precipitation of pure FXD in (C) 0.01 mol/L NaOH media under 200× magnification (the middle figure in the third row), Figure S6-3:PLM images of dissolution and precipitation of pure FXD in (C) 0.01 mol/L NaOH media under 200× magnification (the right figure in the third row).
