# Supplementary material for: Solid Form and Phase Transformation Properties of Fexofenadine Hydrochloride during Wet Granulation Process
Source: Pharmaceutics. 2021 May 27;13(6):802. doi: 10.3390/pharmaceutics13060802 (PMC8229471; doi:10.3390/pharmaceutics13060802)
Supplement: Supplementary file 1 [file pharmaceutics-13-00802-s001.zip › pharmaceutics-1232699-supplementary.pdf]

pharmaceutics

Supplementary Materials: Solid Form and Phase Transformation Properties of Fexofenadine Hydrochloride during Wet Granulation Process

Suye Li, Hengqian Wu, Yanna Zhao, Ruiyan Zhang, Zhengping Wang and Jun Han

Table S1. The comparison data with relevant data published in the literatures or patents.

DSC			
Form type	Literature or Patent	state	Melting range/endothermic peak (°C)
Form I	in this paper	anhydrous	198
Form I	US 5738872	anhydrous	193 -199
Form II	in this paper	monohydrate	128
Form II	US 5738872	hydrate (1:0.1-5)	124 -126
**PXRD**			
Form type	Literature or Patent	Characteristic peaks	
Form I	in this paper	5.9°, 7.5°, 12.1°, 14.2°, 15.0°, 17.9, 18.3°, 20.0° (2θ)/ 14.8, 11.8, 7.3, 6.2, 5.9, 5.0, 4.8, 4.4 Å (d spacings)	
Form I	US 5738872	3.7, 3.8, 3.9, 4.4, 4.8, 5.0, 5.9, 6.3, 7.3, 11.8 Å (d spacings)	
Form II	in this paper	7.7°, 11.2°, 13.7°, 16.9°, 18.1°, 18.5, 19.8°, 21.1° (2θ)/ 11.4, 7.8, 6.5, 5.2, 4.9, 4.8, 4.5, 4.2 Å (d spacings)	
Form II	US 5738872	3.5, 3.6, 3.7, 4.1, 4.2, 4.4, 4.7, 4.9, 5.2, 6.4, 7.8 Å (d spacings)	

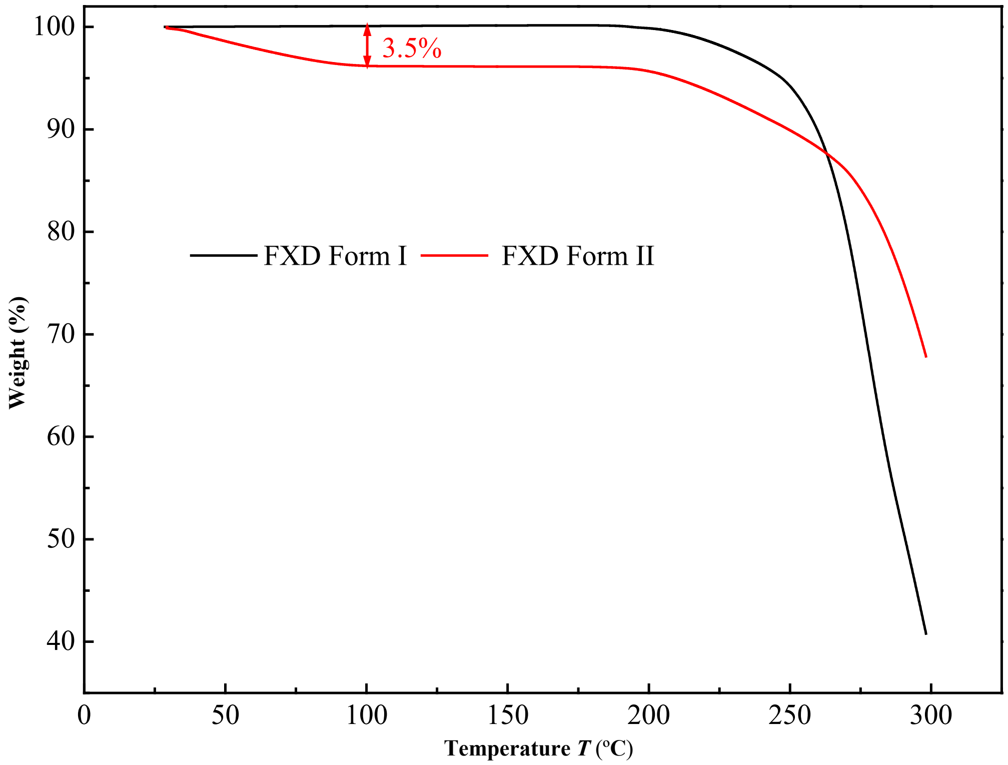

Figure S1. Thermogravimetric analysis of the FXD solid forms.

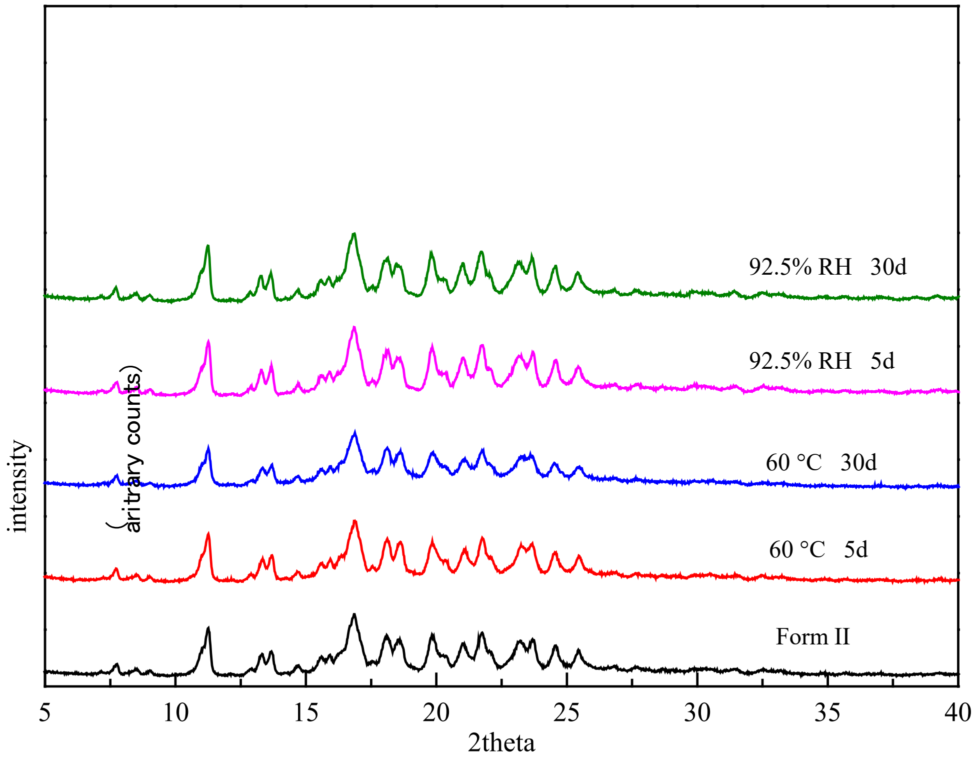

Figure S2. The stability study results of Form II.

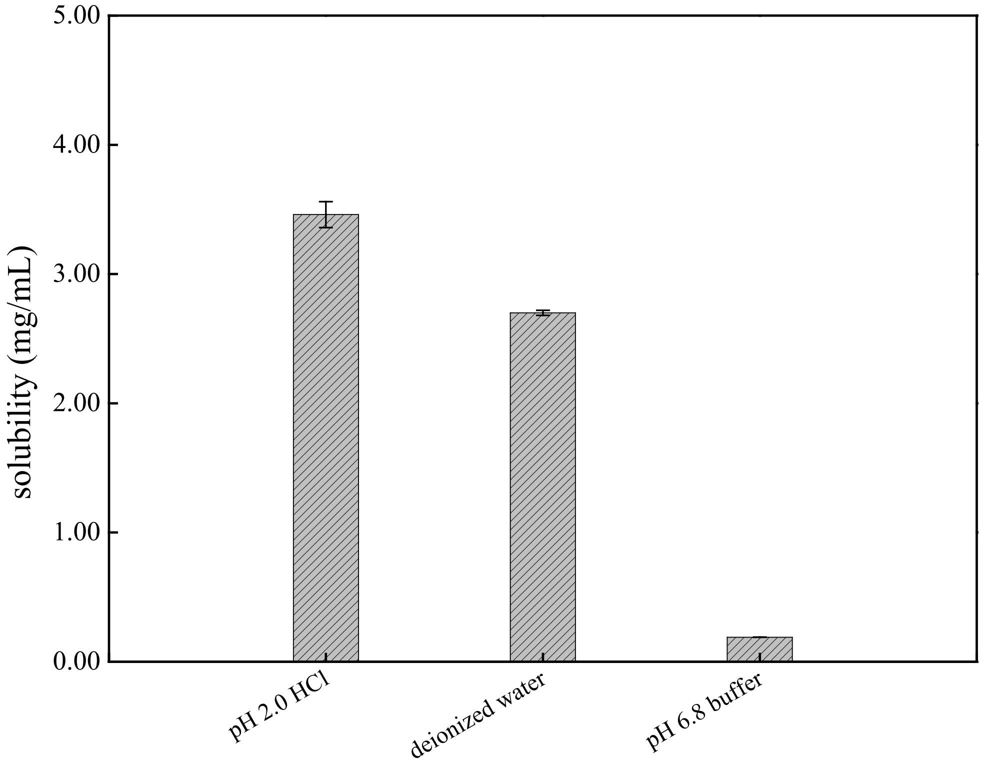

Figure S3. Histogram showing the study of equilibrium solubility of FXD in different media.

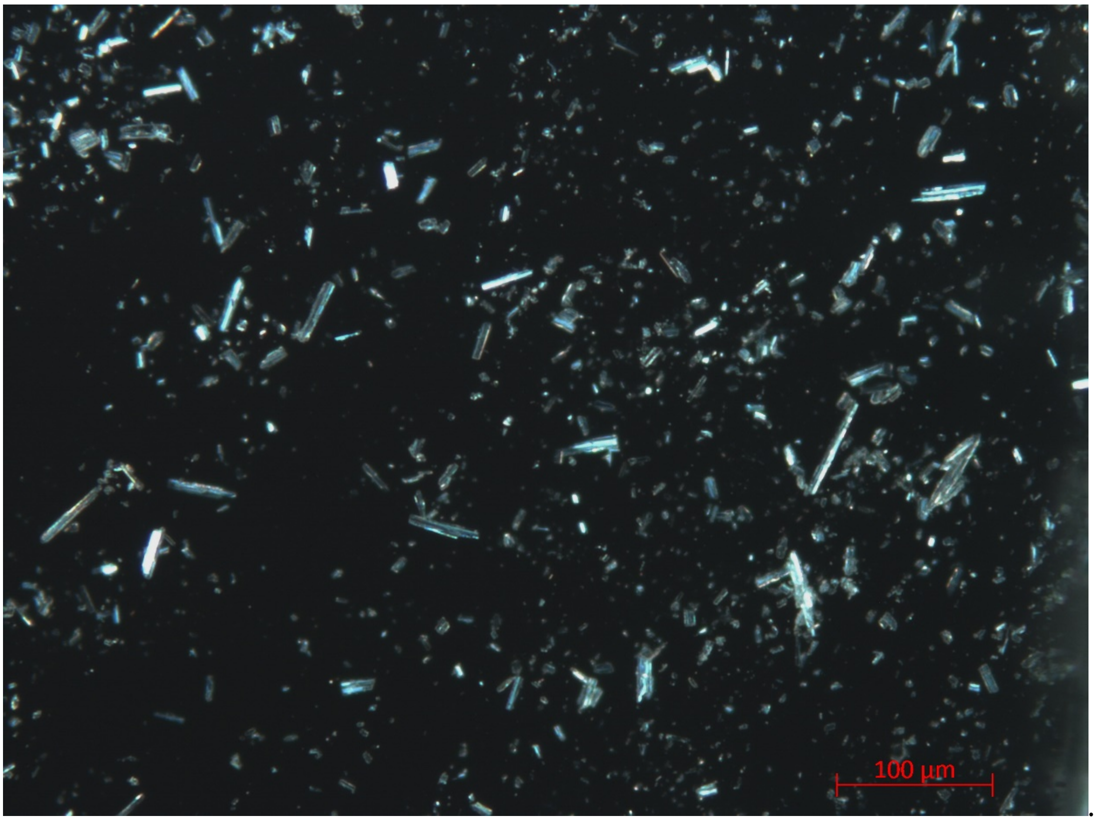

Figure S4-1. PLM images of dissolution and precipitation of pure FXD in (A) 0.01 mol/L HCl media under 200× magnification (the left figure in the first row).

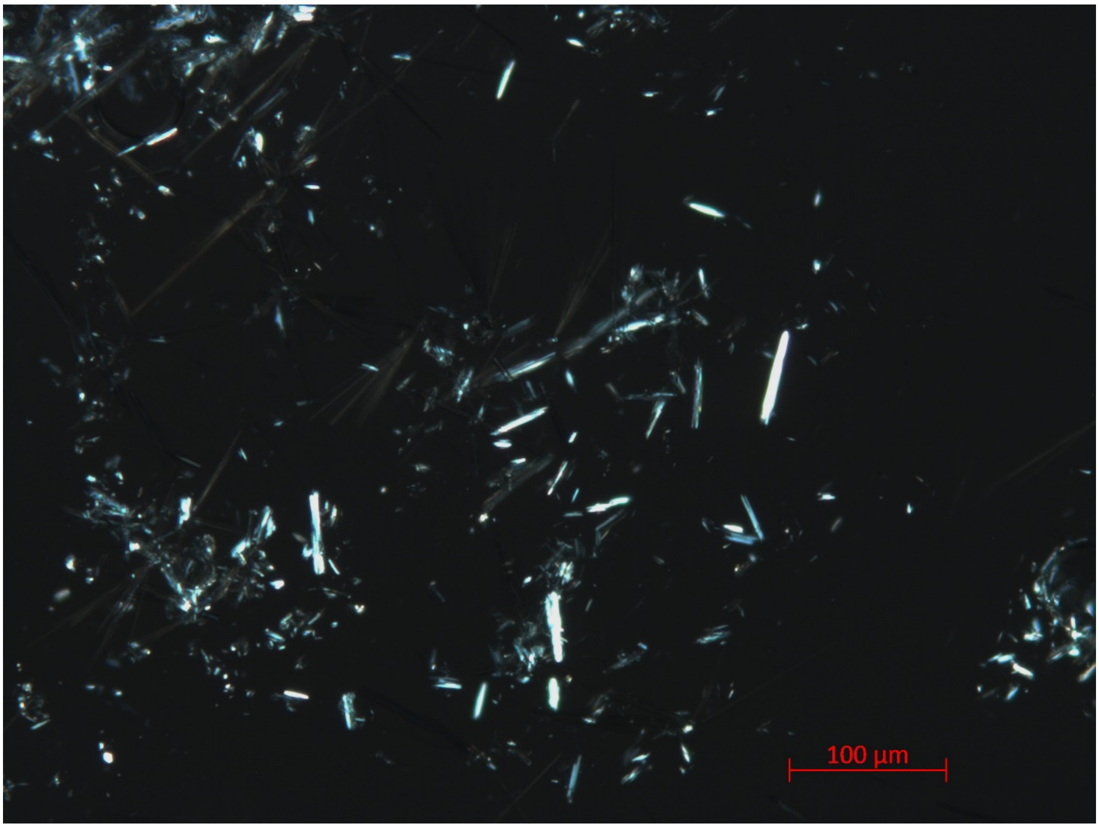

Figure S4-2. PLM images of dissolution and precipitation of pure FXD in (A) 0.01 mol/L HCl media under 200× magnification (the middle figure in the first row).

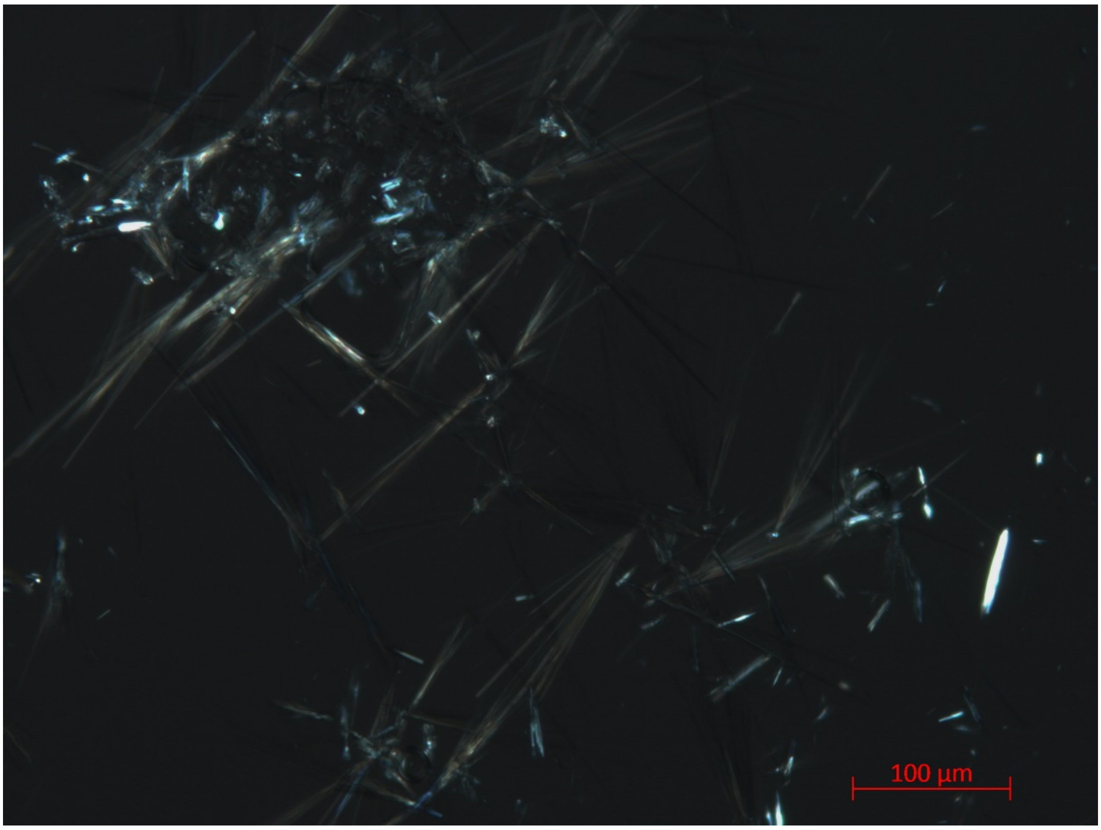

Figure S4-3. PLM images of dissolution and precipitation of pure FXD in (A) 0.01 mol/L HCl media under 200× magnification (the right figure in the first row).

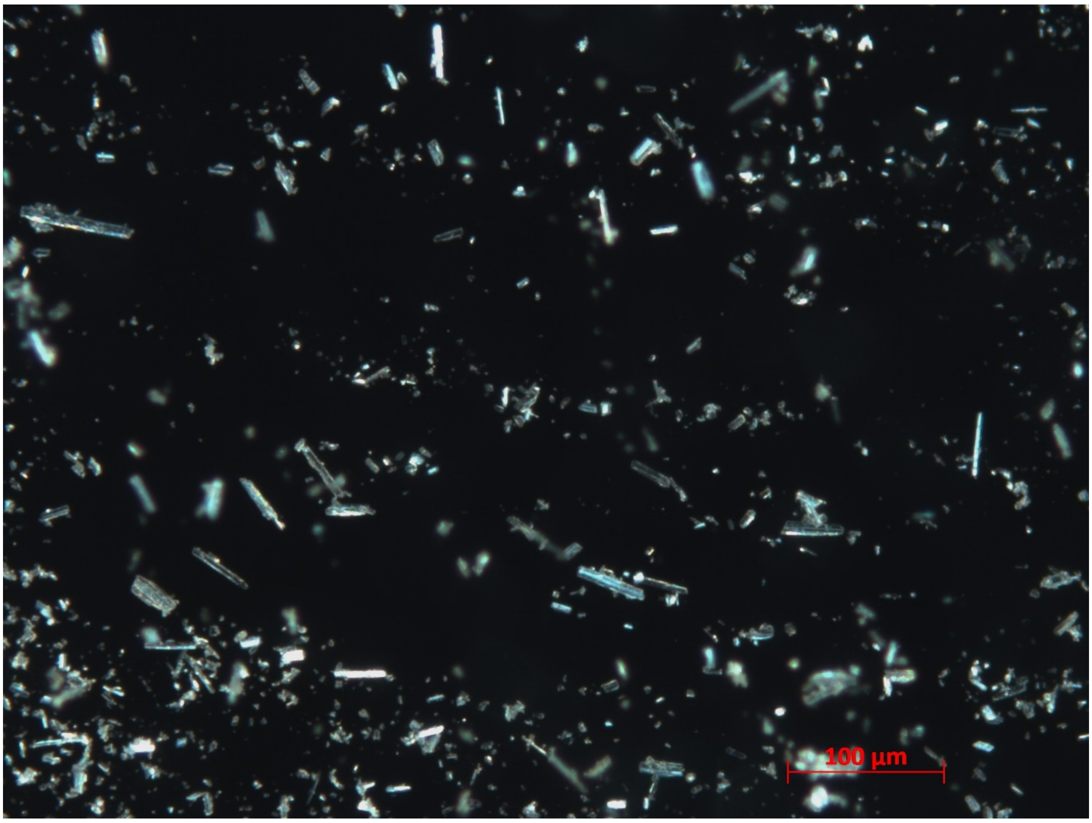

Figure S5-1. PLM images of dissolution and precipitation of pure FXD in (B) pure water media under 200× magnification (the left figure in the second row).

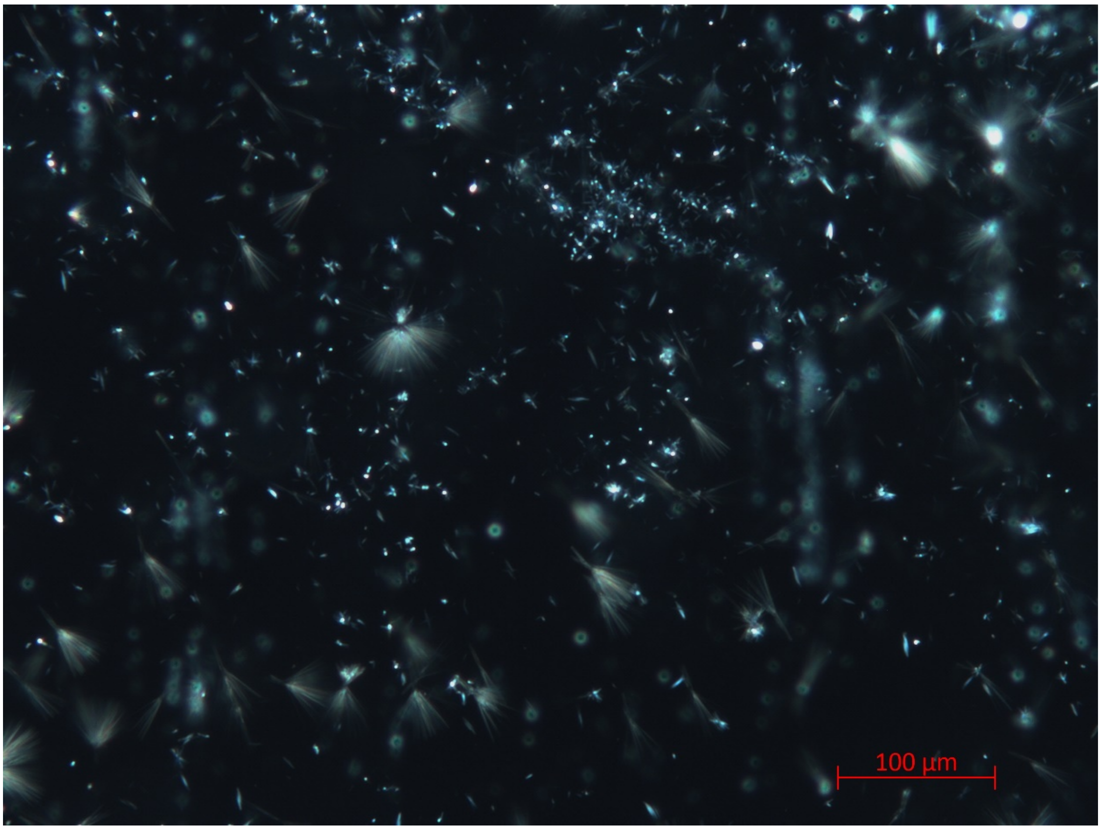

Figure S5-2. PLM images of dissolution and precipitation of pure FXD in (B) pure water media under 200× magnification (the middle figure in the second row).

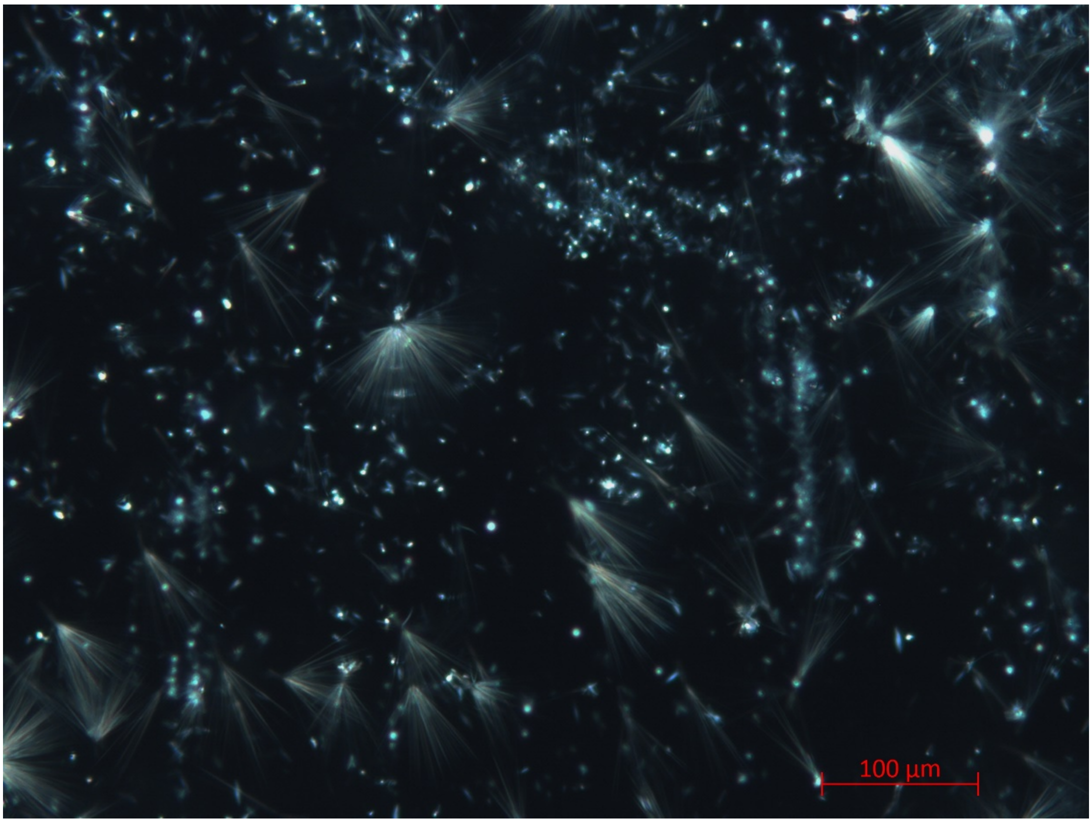

Figure S5-3. PLM images of dissolution and precipitation of pure FXD in (B) pure water media under 200× magnification (the right figure in the second row).

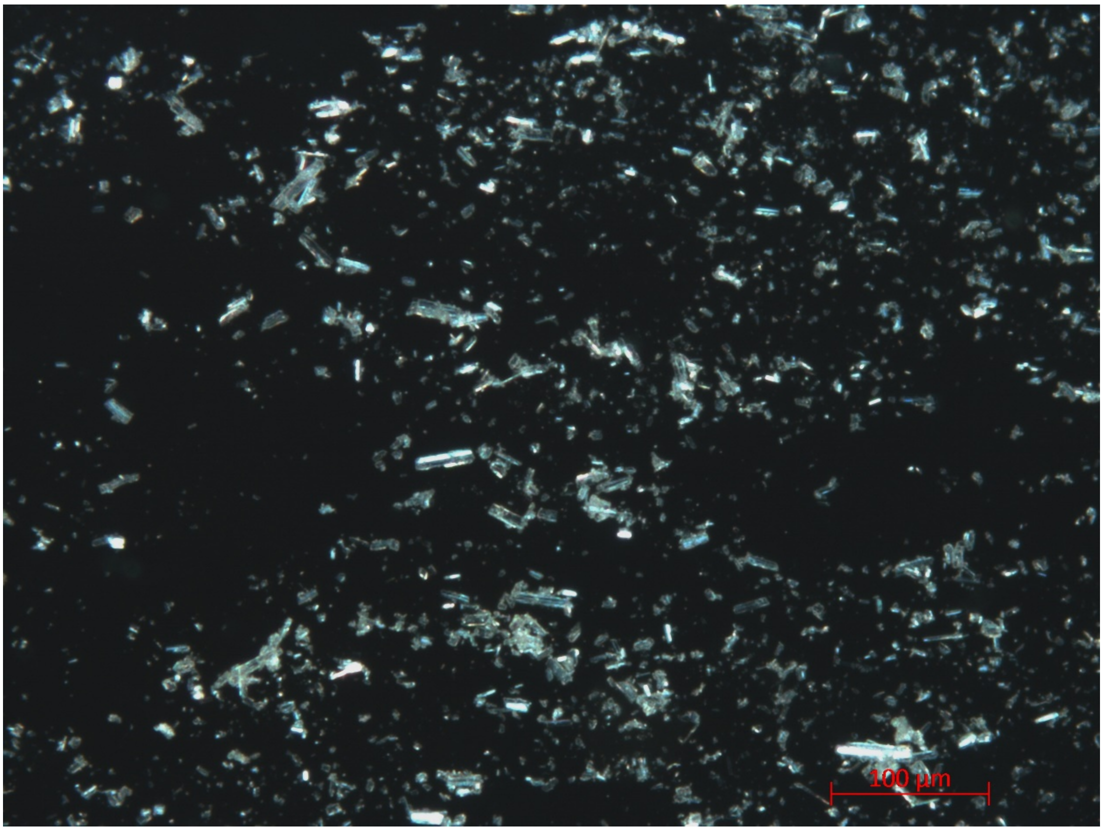

Figure S6-1. PLM images of dissolution and precipitation of pure FXD in (C) 0.01 mol/L NaOH media under 200× magnification (the left figure in the third row).

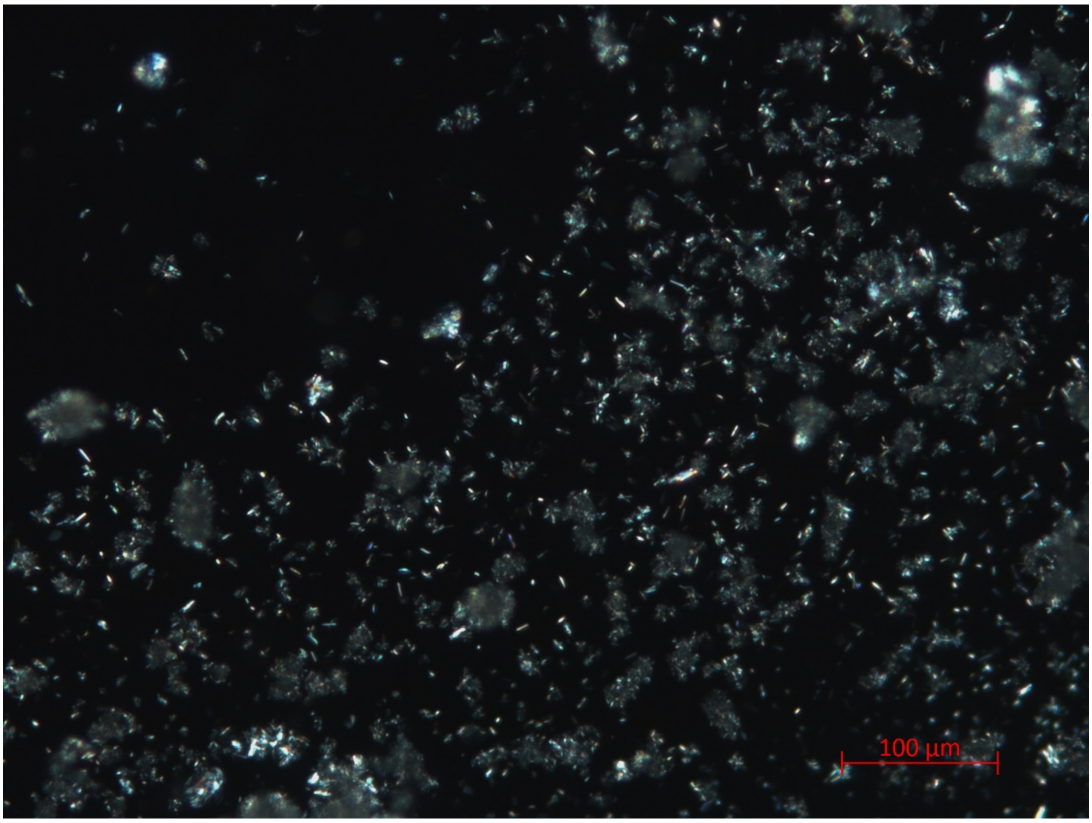

Figure S6-2. PLM images of dissolution and precipitation of pure FXD in (C) 0.01 mol/L NaOH media under 200× magnification (the middle figure in the third row).

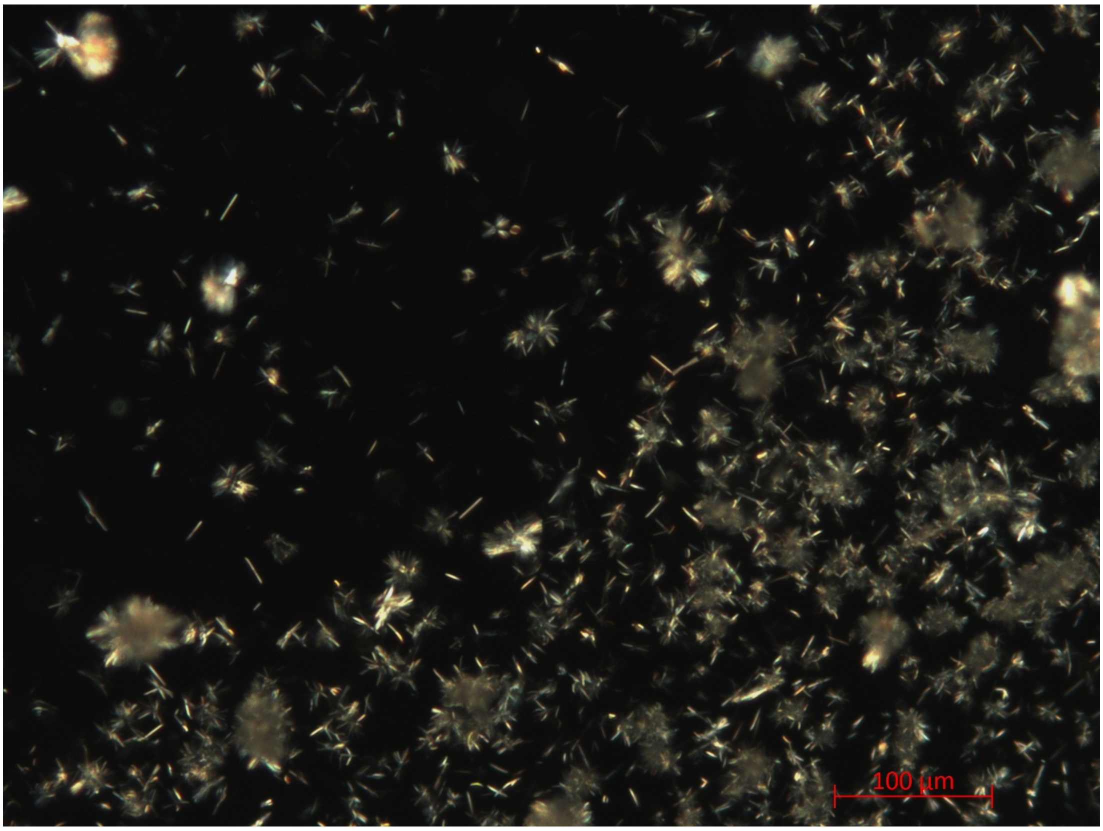

Figure S6-3. PLM images of dissolution and precipitation of pure FXD in (C) 0.01 mol/L NaOH media under 200× magnification (the right figure in the third row).
